# RETRACTED: Rangasamy et al. Sodium Benzoate, a Metabolite of Cinnamon and a Food Additive, Improves Cognitive Functions in Mice After Controlled Cortical Impact Injury. *Int. J. Mol. Sci.* 2022, *23*, 192

**DOI:** 10.3390/ijms27052225

**Published:** 2026-02-27

**Authors:** Suresh B. Rangasamy, Sumita Raha, Sridevi Dasarathy, Kalipada Pahan

**Affiliations:** 1 Division of Research and Development, Jesse Brown Veterans Affairs Medical Center, Chicago, IL 60612, USA; 2 Department of Neurological Sciences, Rush University Medical Center, Chicago, IL 60612, USA

The journal retracts the article “Sodium Benzoate, a Metabolite of Cinnamon and a Food Additive, Improves Cognitive Functions in Mice after Controlled Cortical Impact Injury” [1] cited above.

Following publication, concerns were brought to the attention of the Editorial Office regarding the integrity of a number of images presented in this publication [1].

Adhering to our standard procedure, an investigation was conducted by the Editorial Office and Editorial Board that confirmed multiple overlaps between sub-images within Figure 2 presenting different experimental conditions. While the authors fully collaborated within this process, supporting material and explanations provided did not meet the expectations of the Editorial Board. As a result, the Editorial Board has decided to retract this publication, as per MDPI’s retraction policy (https://www.mdpi.com/ethics#_bookmark30, accessed on 3 February 2026). 

This retraction was approved by the Editor-in-Chief of the *International Journal of Molecular Sciences*.

Authors agree to retract the article.
